# The Influence of Organic Fertilizers on the Abundance of Soil Microorganism Communities, Agrochemical Indicators, and Yield in East Lithuanian Light Soils

**DOI:** 10.3390/plants10122648

**Published:** 2021-12-02

**Authors:** Diana Sivojiene, Audrius Kacergius, Eugenija Baksiene, Aiste Maseviciene, Lina Zickiene

**Affiliations:** 1 Lithuanian Research Centre for Agriculture and Forestry, Vokė Branch of Institute of Agriculture, Žalioji Sq. 2, LT-02232 Vilnius, Lithuania; audrius.kacergius@lammc.lt (A.K.); eugenija.baksiene@lammc.lt (E.B.); 2 Agrochemical Research Laboratory of Institute of Agriculture, Lithuanian Research Centre for Agriculture and Forestry, Savanoriu Av. 287, LT-50127 Kaunas, Lithuania; aiste.maseviciene@lammc.lt (A.M.); lina.zickiene@lammc.lt (L.Z.)

**Keywords:** physiological groups of soil microorganisms, fertilization, agroecosystems

## Abstract

Soil microorganisms are one of the main indicators used for assessing the stability of the soil ecosystem, the metabolism in the soil, and its fertility. The most important are the active soil microorganisms and the influence of the fertilizer applied to the soil on the abundance of these microorganisms. We aimed to investigate how the applied organic fertilizers affect the most active soil microorganisms, which determine the soil fertility and stability. Fungi, yeast-like fungi abundance, and abundance of three physiological groups of bacteria were analyzed: non-symbiotic diazotrophic, organotrophic, and mineral nitrogen assimilating. This study is valuable because relatively few similar studies have been performed on infertile Lithuanian soils. The first results of a long-term study were obtained. The results show the effect of fertilizers on trends in the changes of microorganism community diversity; however, more analysis is needed to assess the impact of organic fertilizers on the most active soil microorganisms. Therefore, the investigation was continued. The results of the 2020 quantitative analysis of culturable soil microorganisms show that the highest abundance of organotrophic and non-symbiotic diazotrophic bacteria were recorded during the summer season. Meanwhile, the abundance of bacteria assimilating mineral nitrogen and fungi was higher in autumn. Agrochemical parameters were determined at the beginning of the experiment. The highest concentration of N_min_ in the soil was determined after fertilizing the plants with the combination of granulated poultry manure (N_170_) + biological substance *Azotobacter* spp. The yield of barley was calculated. It was found that the highest yield of spring barley in 2020 was obtained by fertilizing the experimental field with organic in combination with mineral fertilizers.

## 1. Introduction

On a global scale, only a small part of the planet’s land area is suitable for agriculture; therefore, it is very important to keep the soil fertile [1]. Soil microorganisms play an important role in the process of maintaining soil fertility; they are the most important participants in the transformation (change) of various organic and mineral substances, which determine the yield of plants [2,3].

Intensive fertilization of the soil with mineral fertilizers has a negative effect on the soil biota, and the diversity of microorganism species decreases, which promotes the appearance of niches for the establishment of pathogenic organisms. With the right farming strategy, soil microorganisms enrich and supplement plants with a variety of necessary and available compounds. Without good farming conditions and intensification of agricultural activities, the amount and activity of microorganisms decreases significantly, resulting in poor plants and crop yields. Therefore, with the intensification of agricultural activities, research on the diversity and activity of soil microorganisms is becoming especially important. Research has already shown that greater diversity of microorganism communities has a decisive impact on soil quality [4,5,6,7,8,9].

Fertilization with organic fertilizers increases the mass and diversity of the soil microbiota, which is very important for the decomposition of organic matter and plant nutrition [10,11]. Litter cattle manure enriches soil with organic carbon, increasing its fertility, and binds soil organic aggregates, thus increasing their stability and reducing the degradation of organic matter [12,13]. Recently, the use of granular organic fertilizers, made not only from cattle but also from poultry litter, which is rich in nutrients available to plants, and free of weed seeds and pathogens, is becoming more widespread [14,15,16,17]. The use of animal manure for fertilization is recommended, but its effects on soil microorganism communities have not been definitively elucidated. Some recent studies suggest that pig and chicken manure significantly increases the abundance of soil bacteria by correlating with an increase in total soil nitrogen. Meanwhile, composted pig manure reduced the amount of some organotrophic bacteria [18]. Some research shows the advantage of granular organic fertilizers over ordinary manure [19]. However, single fragmentary studies are not enough. Granular fertilizers can also be produced from sewage sludge, thus solving the problem of using this waste [20].

The specific biocenosis of microorganisms depends primarily on the agrochemical properties of soil. Another important factor is the cultivated agricultural crops, their change, and the use of fertilizers and other chemicals [21,22,23]. The amount of mineral nitrogen (N-NH_4_ and N-NO_3_) in the soil is very important for plant nutrition, which is constantly changing due to the activity of microorganisms, fertilization technologies, precipitation, ambient temperature, granulometric composition, organic matter content, soil moisture, and other factors [24,25,26,27]. The amount of nitrogen in the soil not only determines the yield and quality of agricultural crops, but its excess has a negative impact on the environment. Once in the soil, nitrogen is constantly transformed, and its most dangerous form is nitrates [27,28,29]. The nitrate concentration in the soil can be increased by mineralization processes of organic matter that are too fast, excessive amounts of fertilizers, high environmental and soil temperatures, and slow infiltration properties of the topsoil [25,30,31]. In the presence of excess water (precipitation), nitrogen is easily leached from the soil, mainly in the form of nitrates (N-NO_3_) (90–98%) [28]. In addition, the Nitrates Directive (91/676EEC) [32] obliges and promotes the protection of European Union waters against nitrate pollution. Therefore, it is very important to know how the amounts of mineral nitrogen in the soil change, not only due to different organic fertilizers, but also how environmental factors can affect it, with the highest probability of nitrogen leaching.

Studies of soil microorganisms, due to their high abundance in a small volume of soil, are quite difficult but very important in terms of ecosystem stability, soil metabolism, and fertility [8,33,34]. The most active soil microorganisms are the most important in this case, as well as the influence of fertilizers applied to the soil on these microorganisms.

Studies shows that low rates of mineral fertilizers have a positive effect on plant productivity and soil agrochemical and microbiological properties. Mineral fertilizers stimulate the growth of ammonifying and nitrifying microorganisms, as well as the proliferation of spore-forming bacteria and stimulates the mineralization of crop residues [35,36,37]. However, the use of mineral fertilizers to increase fertility leads to complete soil degradation. Long-term scientific research by Gautam and co-authors has shown a clear advantage of organic over mineral fertilization [38].

Scientists use a variety of methods to determine the composition, diversity, and abundance of soil microorganism communities. The estimates of the abundance of microorganisms performed by the method of inoculation and cultivation allow the typical presence of active microorganism populations in the soil to be revealed, which, in fact, determine the condition and productivity of the soil [39,40].

This long-term experiment investigates for the first time the effects of new rates of granular poultry and cattle manure fertilizers and their combinations with bioadditives on soil microbiological activity, ecological condition, soil sustainability, and crop productivity. The initial microbiological studies discussed in this article are one of the contributions to the overall long-term study. Therefore, the aim of these studies was to determine the influence of different organic fertilizers and their combinations with biological additives on soil microbiota and the mineral nitrogen concentration in soil, as well as the spring barley yield.

## 2. Results and Discussion

### 2.1. Abundance of Different Physiological Groups of Soil Microorganisms

**Figure 1:**
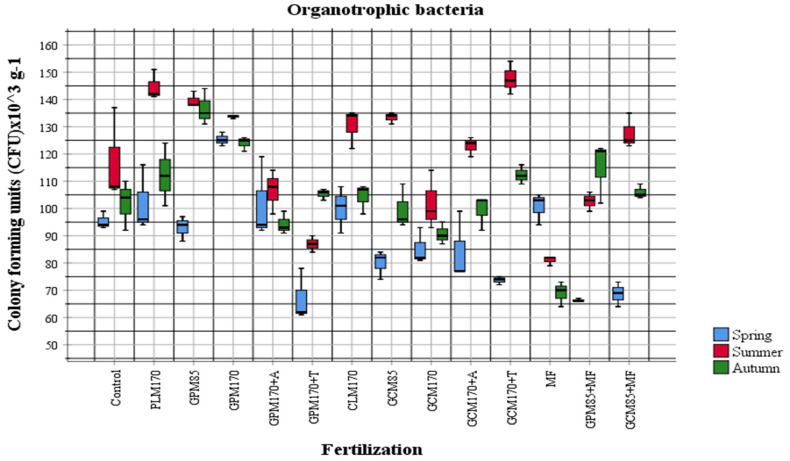
The abundance of organotrophic bacteria in 2020 (*p* < 0.05).

**Figure 2:**
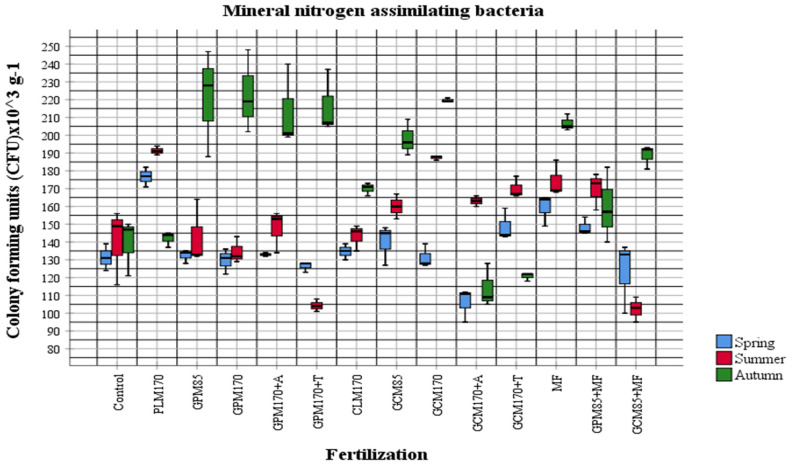
The abundance of mineral nitrogen-assimilating bacteria in 2020 (*p* < 0.05).

**Figure 3:**
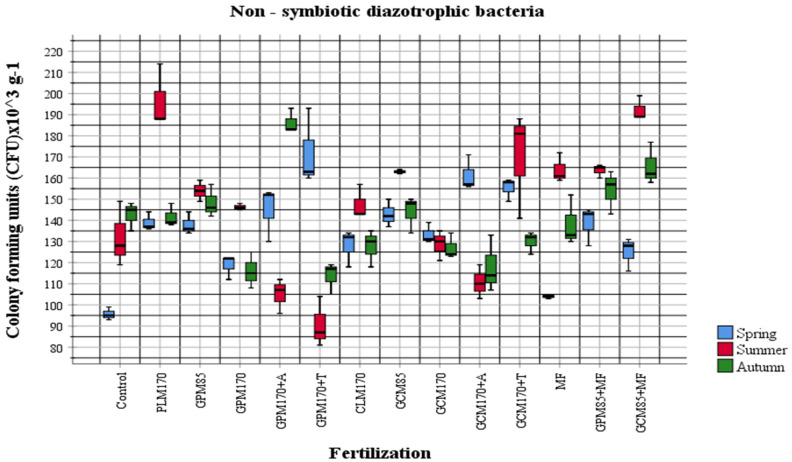
The abundance of non-symbiotic diazotrophic bacteria in 2020 (*p* < 0.05).

**Figure 4:**
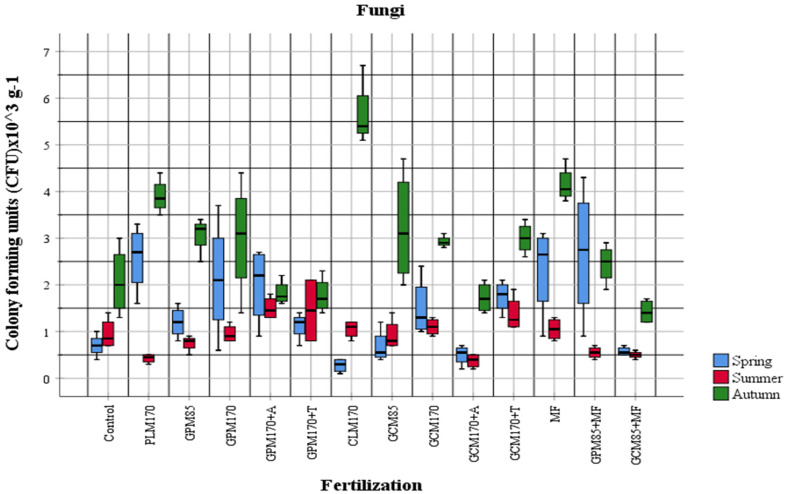
The abundance of fungi in 2020 (*p* < 0.05).

In order to evaluate the influence of fertilizers applied to the soil on the abundance of physiological groups of most active soil microorganisms, the obtained quantitative data were compared according to different fertilization options. Organic fertilizers were applied to the soil in the autumn of the first year of investigation in 2018 and mineral fertilizers were applied to the soil in 2020, before pre-sowing tillage (ploughing). The abundance and changes in the amount of different physiological groups of cultivable soil microorganisms in the plant vegetation period in 2020 were observed during the quantitative analysis (Figure 1, Figure 2, Figure 3 and Figure 4).

Studies by other authors have shown that fertilization with organic fertilizers initially increases the amount of ammonifiers (organotrophic bacteria), and later, especially at the end of the growing season, the amount of nitrifiers (mineral nitrogen-assimilating bacteria) increases [41]. Otherwise, if the abundance of nitrifiers is high in the spring, then nitrate depression is possible [42], while abundant mineral fertilization reduces the amount of nitrifiers.

The results of our investigations showed that in general, the abundance of organotrophic bacteria was highest in the summer period. Comparing the data of individual samples with the control, we see that the proportion of organotrophic bacteria was increased in all samples fertilized with manure, except for the samples with *Trichoderma* and mineral fertilizers. As the manure was added in the autumn of 2018, an increase in organotrophs was to be expected, but the environmental conditions for active manure processing were not favorable. In both 2019 and 2020, temperatures were high, but a lack of moisture was felt. In the spring of 2020, dried manure was observed during tillage. The abundance of diazotrophs was different in different areas compared to the control variant (GPM_170_ + A, GPM_170_ + T) and showed a significant decrease during the summer (Figure 3). At that time, the amount of diazotrophs in the GPM + T sample was quite high in the spring (Figure 3). When manure is used for fertilization, an increase in organotrophs is usually observed first, followed by an increase in the abundance of nitrifiers, and an overall increase in soil activity is also observed [43,44]. Fertilization of manure also affects the taxonomic composition of microorganisms and increases the number of more active bacteria belonging to the taxonomic groups of *Actinobacteria* and *Proteobacteria*, which can grow in the nutrient medium [43,44,45]. In our studies, a significant increase in the abundance of nitrifiers was observed in samples fertilized with poultry manure in the autumn (Figure 1). The highest abundance of the fungal communities was also determined in autumn (2.87 ± 1.24 × 10^3^ CFU g^−1^). During the summer period, the representatives of this group were determined to be the lowest (0.93 ± 0.44 × 10^3^ CFU g^−1^). According to the microscopic and cultural features, predominant cultures strongly related to the genera *Trichoderma, Paecilomyces, Cladosporium, Fusarium, Aureobasidium, Colletotrichum, Trichothecium, Penicillium, Saccharomyces, Mortierella,* and *Phiallophora* were identified.

The data of our study demonstrate that mineral nitrogen-assimilating bacteria were most abundant in the MF-fertilized test field. Organotrophic bacteria were more abundant in the test field fertilized with granulated poultry manure, non-symbiotic diazotrophic bacteria in the granulated cattle manure combined with mineral fertilizers-fertilized field, and fungi in the MF-fertilized field.

### 2.2. Investigations of Mineral Nitrogen in Soil

Proper and timely use of organic fertilizers improves plant growth and development, and it is necessary for the formation of a humus layer in the soil. Lately, in addition to manure, i.e., organic fertilizers, granulated manure and other various liquid humic, biohumus, or plant extracts are increasingly used more frequently. Nitrogen is released from different types of organic fertilizers into plant uptake compounds with varying intensity; therefore, it is important to study the release of nutrients in the soil and their uptake.

Prior to the experiment, soil pH_KCl_ was rather acidic (5.7–6.0), mobile phosphorus (P_2_O_5_) was very high (202–249 mg kg^−1^), mobile potassium (K_2_O) was average (118–137 mg kg^−1^), and mineral nitrogen (N_min_) and mineral sulphur (S_min_) 0–60 cm were average (8.11 and 2.69 mg kg^−1^ soil, respectively).

The concentrations of mineral nitrogen (N_min_) in the soil were mainly determined by fertilization of winter rye with organic fertilizers in autumn. Therefore, in the spring of 2019, higher concentrations of N_min_ were accumulated in the soil (Table 1) compared to the previous autumn, when the above-mentioned indicator averaged 8.11 mg kg^−1^ before the establishment of the experiment, and only in the control and MF fields, the N_min_ concentration was lower. The evaluation of the effectiveness of different organic fertilizers demonstrated that in the 0–60 cm layer of soil, a higher content of these plant nutrients was found when fertilizing plants with both loose and granulated poultry manure fertilizers and combinations with biological substances than in fields fertilized with cattle manure. The concentrations of N_min_ in the soil ranged from 12.50 to 15.68 mg kg^−1^ when fertilized with different rates and different forms of poultry manure, and with cattle manure from 7.98 to 11.57 mg kg^−1^. The highest (15.68, 13.96, and 13.73 mg kg^−1^) N_min_ concentrations were determined after the application of GPM_170_ + A and GPM_170_ + T and GPM_170_ or GPM_85_ + MF to the soil. When fertilizing plants with granulated cattle manure, it was most effective in combination with a biological substance: GCM_170_ + A.

In the spring of 2020, the N_min_ concentration in the soil was significantly less than in 2019. These low N_min_ concentrations were largely due to the lighter particle size distribution of the soil and the warmer than usual meteorological conditions during the winter-spring period, when in the absence of freezing, soil and rain N_min_ leaches from the top layers to the deeper layer, and eventually to the groundwaters. These trends have been confirmed by other researchers. Swedish researchers claim that nitrogen leaching is increased by warm winters, higher rainfall, lighter soils, and higher organic matter [46]. According to research by Rutkowska and Fotyma (2011) [47], the amount of mineral nitrogen in the soil is highly dependent on the granulometric composition of the soil. Žičkienė (2016) [48] also found that the concentration of mineral nitrogen in the 0–60 cm soil layer in spring depended mostly on the average precipitation and average air temperature (November–March) in soils with a light granulometric composition. Therefore, due to the prevailing soil moisture regime in the country, higher precipitation during the winter is likely to affect nitrate leaching into deeper soil layers, resulting in the low N_min_ levels in the soil in the spring of 2020. In addition, the effectiveness on the N_min_ concentration of both litter and granulated poultry and cattle manure fertilizers applied two years ago was not determined either. Irrespective of pre-sowing fertilization with organic fertilizers, the content of mineral nitrogen in the 0–60 cm soil layer was very low and varied from 1.29 mg kg^−1^ in the control plot, and from 1.60 to 2.31 mg kg^−1^ in the fertilized plot. Slightly higher N_min_ concentrations were determined only when fertilizing plants with GPM_170_, GPM_170_ + A, and GPM_85_ + MF. According to various authors, the use of composted or ordinary manure for fertilization prevents the active leaching of nitrogen from the soil, especially sandy loam [49,50,51]. In general, the use of biochar not only increases the nitrogen content in the soil, and initiates the activity of microorganisms, but also prevents nitrogen leaching [52].

The evaluation of the efficiency of biological substances for the mentioned indicator demonstrated that in 2020 of the investigation, only the biological substance with the nitrogen-fixing bacterium *Azotobacter* spp. (No 1) showed a tendency to increase the concentration of N_min_ in the soil (Table 1). It worked effectively when used in combination with both GPM_170_ and GCM_170_. The effect of the biological substance containing the fungus *Trichoderma* spp. (No 2) was less regular, and in 2020, when used in combination with both GPM_170_ and GCM_170_, less N_min_ was found in the soil than when fertilizing with granulated poultry or cattle manure at the rate of 170 kg ha^−1^.

Summarizing the results of the two-year study, it can be seen that the concentration of mineral nitrogen depends not only on the fertilization with organic fertilizers, but also on the meteorological conditions in the winter-spring period. The effectiveness of organic fertilizers compared to unfertilized plants became apparent only in the first year of the investigation after their application. The evaluation of the efficiency of the biological substances showed that the biological substance with the nitrogen-fixing bacterium *Azotobacter* spp. (No 1) tended to increase the N_min_ concentration in soil when used in combination with both GPM_170_ and GCM_170_. According to Schlegel and coauthors, organic manure contains many macro and trace elements and organic matter that improve the chemical and physical properties of the soil and the microbial biomass, and at the same time increase crop yields [53]. It has also been found that the use of chicken manure fertilizers can maintain a stable amount of nutrients in the soil and reduce the release of mineral fertilizers into the environment [54].

### 2.3. Spring Barley Yield

The evaluation of the 2020 barley yield results demonstrated that the highest yield supplement was obtained by using GPM_85_ + MF for soil fertilization, as well as GCM_85_ + MF and MF. The higher yield is due to the fact that mineral fertilizers were applied to the soil in 2020, before pre-sowing cultivation (ploughing), and organic fertilizers in the autumn of the first year of the investigation (2018). Similarly, a higher straw yield and a maximum 1000-grain weight from the correspondingly fertilized soil was observed. The 2020 barley yield results are presented in Table 2. Several researchers have also found that mineral fertilizers increase crop yields more than granular organic (OGF) or granular organic fertilizers with mineral additives (OMF). Nutrients from OGF and OMF are released at a slower rate and can be regarded as a delayed release [16,55]. Therefore, it is likely that slower nitrogen release allows nutrients to be retained in the soil for longer, so plants use them throughout the growing season, which can also have a positive effect on their yield. Additionally, Correa and co-authors observed that the use of organo-mineral fertilizers with inhibitors increased the yield of corn and wheat crops compared to organic fertilizers [56].

## 3. Materials and Methods

### 3.1. Experimental Treatments and Design

Quantitative research of soil microorganisms was performed in the Lithuanian Research Centre for Agriculture and Forestry in the laboratory of the Vokė branch of Institute of Agriculture. The soil samples were taken from the field of a long-term experiment started in the autumn of 2018. The soil of the experimental site is basic illimerised soil (according to FAO, the soil of the experimental site is *Sandy Loam Haplic Luvisol*). The total area of the experimental field is 24 m^2^ (6 × 4 m), and the accounting area is 15 m^2^ (5 × 3 m). The field layout was randomized, with four replications.

The two most used types of organic fertilizers for agricultural crops were selected for the investigation litter and granulated (poultry and cattle) manure. These fertilizers differ in the amount of nutrients (concentration) and the intensity of nutrient release. Organic and mineral fertilizers were used in the field experiment: poultry litter manure (PLM), peat cattle litter manure (CLM), granulated poultry manure (GPM), granulated cattle manure (GCM), and mineral fertilizers (MFs) (ammonium nitrate (34.4% N), granulated superphosphate (19.0% P_2_O_5_; 13.0% S), and potassium chloride (60.0% K_2_O)). The abbreviations are explained in Table 3.

Organic fertilizers were applied to the soil in the autumn of the first year of the investigation (2018), for winter rye, and in the third year, in autumn 2020, before growing potatoes in 2021. The rate of organic fertilizer was calculated based on 170 kg ha^−1^ of nitrogen active substance. Mineral fertilizers for winter rye were applied after the regeneration of plant vegetation, and for the remaining crop rotation plants, before pre-sowing cultivation (ploughing). Winter rye and potatoes were fertilized at the N_90_P_60_K_90_ mineral fertilizer rate, and spring barley and spring wheat at N_60_P_60_K_60_.

A non-commercial biological additive was used in the experiment: nitrogen-fixing bacteria *Azotobacter* spp. (No 1)—A (a mixture of two cultures: *A. chroococcum* and *A. vinelandii* together with the residues of the culture medium), microelements (manganese, iron, copper, molybdenum, zinc, and cobalt), and vitamins (B1, B3, B6), no more than 0.02%; biological substance containing the fungi *Trichoderma* spp. (No 2), T. enriched with phytohormones (a mixture of three cultures: *T. harzianum, T. tomentosum*, and *T. viride*). Biological substances were sprayed annually in spring for all crops on the soil prior to sowing or until the plants completely covered the soil surface, except for winter rye.

The experiment was performed in the following crop rotation order: winter rye (2018/2019), spring barley (2020), potatoes (2021), spring wheat (2022), and winter rye (2023/2024). The preceding crop was buckwheat.

### 3.2. Soil Sampling and Microbial Count

The prevalence of different physiological groups of cultivable soil microorganisms was determined in soil samples of natural moisture. The soil samples were taken from a 10–20 cm deep arable layer from the four replicates of the relevant test fields and a pooled sample was formed from four replicates of each sample. For the quantitative analysis of microorganisms, samples were taken three times during the vegetation period (spring, summer, and autumn) and performed using the plate count technique by inoculating the diluted soil suspension on appropriate agar media [57]: for micromycetes and yeasts, Sabouraud agar with chloramphenicol (200 ppm) (Liofilchem, Italy); for organotrophic bacteria, meat peptone nutrient agar (Liofilchem, Italy); for mineral nitrogen-assimilating bacteria, starch-ammonium agar [58]; and for atmospheric nitrogen-fixing bacteria (diazotrophs), Ashby’s mannitol agar [59]. Serial dilutions were prepared to obtain 10^−2^ and 10^−3^ soil suspension, and then 0.1 mL of each dilution were spread on certain nutrient agar in Petri plates with five replicates. Petri dishes with inoculated samples were cultivated at 25 °C in the dark for 7–12 days. The content of microorganisms in colony-forming units (CFU) per 1 g of dry soil was calculated according to the Carter and Gregorich methodology [60]. Fungal colonies on agar were examined microscopically and predominant genera according to handbooks and manuals were identified [61,62,63,64,65,66].

### 3.3. Soil Agrochemical Analysis

The analyses of the soil agrochemical properties were performed before and after the test installation the investigation.

Their methods, depths, and time are presented in Table 4. After collection, soil samples were air-dried or oven-dried at a temperature no higher than 40 °C. Then, soil samples were milled, passed through a 2 mm sieve, and stored in an airtight room in a soil sample storage rack.

The determination of soil pH was performed using 1:5 (vol/vol) soil suspension in 1 M KCl. The mixture was shaken for 60 min and left to sit for 1 h. The pH of the suspension was measured at 20 ± 2 °C stirring with a pH-meter.

Soil mobile phosphorus P_2_O_5_ and mobile potassium K_2_O were extracted using 1:20 (wt/vol) soil suspension of ammonium lactate-acetic acid extractant (pH 3.7). The suspension was shaken for 4 h. Mobile P_2_O_5_ was determined in extract using ammonium molybdate via the spectrometric method with a Shimadzu UV 1800 spectrophotometer. Mobile K_2_O was determined using flame emission spectroscopy with a JENWAY PFP7 flame photometer.

Mineral nitrogen (N_min_) was extracted in 1:5 (wt/vol) soil suspension of 1 M KCl solution. The suspension was shaken for 60 min at 20 ± 2 °C. After shaking, the suspension was filtrated and analyzed using a flow injection analysis (FIA) system by an FIASTAR 5000 analyzer. N_min_ was calculated by adding the sum of nitrate and nitrite nitrogen with ammonia nitrogen.

Mineral sulphur (SO_4_-S) was extracted in a suspension of 1 M KCl solution. The suspension was shaken for 25 min at 20 ± 2 °C. After shaking, the suspension was filtrated, then precipitated with BaCl_2_ solution and analyzed using a spectrophotometer Shimadzu UV 1800.

### 3.4. Plant Yield Evaluation

The barley grain yield was determined from each plot by weighing it at harvest. Before barley was harvested, samples were taken by cutting the bottom parts of plants from 4–5 places in each plot separately. The weight of 1000-grain and grain-straw ratio were determined from the cuttings. The straw yield was calculated according to the determined ratio.

### 3.5. Meteorological Conditions

The meteorological conditions are described on the basis of the data recorded at the station of Lithuanian Hydrometeorological Service under the Ministry of Environment in Trakų Vokė. The daily air temperature during the study period (April September) in 2019 was 9.0–21.1 °C. In 2020 (April September), it was 6.6–19.4 °C. The average precipitation during the study period (April September) in 2019 was 1–100 mm. In 2020 (April September), it was 6–78 mm.

The average precipitation and daily air temperature during the study period are presented in Table 5.

Compared with the average perennial temperature, the 2019 average air temperature (8.8 °C) was 1.9 °C higher than the average perennial air temperature (1981–2010 average), and the 2020 average air temperature (9.2 °C) was 2.3 °C higher than the average perennial air temperature. Annual precipitation in 2020 in Lithuania was 646 mm (7%) less than the perennial norm (694 mm). Both years were very warm but lacked moisture, especially in 2019.

### 3.6. Statistical Analysis

Data analysis was performed using the computer program SPSS. The Duncan Multiple range Test using a 95% (*p* < 0.05) probability level was used for the evaluation.

## 4. Conclusions

Since the soil under study is part of a long-term experiment that is still in progress, it is too early to draw some categorical conclusions. However, trends in the changes of microorganism community diversity can be predicted. As environmental conditions have been markedly sub-optimal, caution should be exercised in assessing the abundance of different physiological groups. Therefore, assessing the total abundance of microorganisms, it can be stated that the abundance of organotrophic and diazotrophic bacteria was highest during the summer period, while nitrifying bacteria and fungi were highest in the autumn. This can be attributed to the significant decrease in nitrogen in the soil in the spring of 2020.

Comparing the research data with the control variants, we can state that the used different types of manure affect the abundance of most active soil microorganisms, but due to the standardization of the manure ration, it is not possible to provide a final answer.

The investigations of the mineral nitrogen in the soil in a stationary field experiment demonstrated that the concentration of mineral nitrogen depends not only on fertilization with organic fertilizers, but also on the meteorological conditions in the winter-spring period. The effectiveness of organic fertilizers compared to unfertilized plants became apparent only in the first year of the investigation after their application. The highest concentration of N_min_ in the soil was determined by fertilizing the plants with the combination of GPM_170_ + A. The evaluation of the efficiency of the biological substances showed that the biological substance with the nitrogen-fixing bacteria *Azotobacter* spp. (No 1) tended to increase the N_min_ concentration in the soil when used in combination with both GPM_170_ and GCM_170_.

The highest grain and straw yield were recorded in cases when the soil was fertilized with one mineral fertilizer and while adding mineral fertilizer rates to the granulated poultry manure.

## Figures and Tables

**Table 1:** Concentration of mineral nitrogen in the 0–60 cm soil layer in spring.

Fertilization Variants	Mineral Nitrogen (N_min_) Concentration 0–60 cm Layer mg kg^−1^ ± Standard Deviation (SD)	
	2019 Spring	2020 Spring
C	6.92 ± 0.41	1.29 ± 0.33
MF	6.71 ± 1.26	1.60 ± 0.38
PLM_170_	13.13 ± 1.13	1.78 ± 0.40
GPM_85_	12.50 ± 0.46	1.64 ± 0.27
GPM_170_	13.73 ± 1.69	2.09 ± 0.47
GPM_170_ + A	15.68 ± 0.56	2.31 ± 0.40
GPM_170_ + T	13.96 ± 1.57	1.80 ± 0.26
CLM_170_	7.98 ± 1.29	1.90 ± 0.30
GCM_85_	7.66 ± 0.11	1.61 ± 0.39
GCM_170_	9.13 ± 1.53	1.88 ± 0.42
GCM_170_ + A	11.57 ± 2.74	1.99 ± 0.42
GCM_170_ + T	9.95 ± 1.71	1.77 ± 0.26
GPM_85_ + MF	13.42 ± 0.78	2.02 ± 0.29
GCM_85_ + MF	9.51 ± 0.37	1.84 ± 0.25

**Table 2:** Spring barley harvest (2020).

Fertilization Variants	Clean, 15% Humidity Grain Yield t/ha ± Standard Deviation (SD)	Grain-Straw Ratio	Straw Yield t/ha ± Standard Deviation (SD)	1000 Grain Weight g ± Standard Deviation (SD)
C	1.20 ± 0.04	1:1.01	1.21 ± 0.13	38.2 ± 1.68
MF	2.72 ± 0.20	1:0.98	2.67 ± 0.47	46.4 ± 1.34
PLM_170_	1.49 ± 0.30	1:0.90	1.33 ± 0.19	41.0 ± 0.38
GPM_85_	1.30 ± 0.35	1:0.99	1.30 ± 0.40	38.1 ± 1.98
GPM_170_	1.26 ± 0.11	1:0.98	1.23 ± 0.12	39.7 ± 1.61
GPM_170_ + A	1.22 ± 0.13	1:0.95	1.15 ± 0.09	40.5 ± 1.41
GPM_170_ + T	1.31 ± 0.14	1:0.95	1.24 ± 0.12	39.3 ± 0.52
CLM_170_	1.48 ± 0.08	1:0.89	1.31 ± 0.11	39.7 ± 2.06
GCM_85_	1.10 ± 0.04	1:1.03	1.13 ± 0.06	38.5 ± 0.86
GCM_170_	1.21 ± 0.04	1:1.06	1.28 ± 0.16	37.7 ± 2.43
GCM_170_ + A	1.14 ± 0.02	1:1.04	1.18 ± 0.07	37.9 ± 2.12
GCM_170_ + T	1.08 ± 0.03	1:1.03	1.12 ± 0.05	37.7 ± 1.48
GPM_85_ + MF	3.15 ± 0.02	1:0.97	3.04 ± 0.32	46.7 ± 0.94
GCM_85_ + MF	3.00 ± 0.02	1:0.99	2.96 ± 0.41	45.9 ± 0.58

**Table 3:** Explanation of abbreviations.

Abbreviation	Explanation
C	control (without fertilizer—N_0_P_0_P_0_)
MF	mineral fertilizers (N_90/60_ *)
PLM_170_	poultry litter manure (N_170_)
GPM_85_	granulated poultry manure (N_85_)
GPM_170_	granulated poultry manure (N_170_)
GPM_170_ + A	granulated poultry manure (N_170_) + biological substance No 1 (*Azotobacter* spp.)
GPM_170_ + T	granulated poultry manure (N_170_) + biological substance No 2 (*Trichoderma* spp.)
CLM_170_	peat cattle litter (N_170_)
GCM_85_	granulated cattle manure (N_85_)
GCM_170_	granulated cattle manure (N_170_)
GCM_170_ + A	granulated cattle manure (N_170_) + biological substance No. 1 (*Azotobacter* spp.)
GCM_170_ + T	granulated cattle manure (N_170_) + biological substance No. 2 (*Trichoderma* spp.)
GPM_85_ + MF	granulated poultry manure (N_85_) + mineral fertilizers (N_90/60_ *)
GCM_85_ + MF	granulated cattle manure (N_85_) + mineral fertilizers (N_90/60_ *)

* N_90_ kg ha^−1^ nitrogen fertilizer rate—winter rye and potatoes, and N_60_—spring barley and spring wheat.

**Table 4:** Methods for analysis of the soil agrochemical properties.

Indicators	Investigation Method	Sampling Time and Frequency, Depth cm	Sources
pH	1 M KCl extraction by potentiometric method	Samples (pH, mobile P_2_O_5_, mobile K_2_O) are taken in the fall before the experiment is set up (2018) and after the experiment is completed (2023). Sampling depth—0–20 cm.	ISO 10390:2005
Mobile phosphorus (P_2_O_5_)	By Egner–Riehm–Domingo (A–L) method in a buffer solution (pH 3.7) extraction (spectrophotometer)		[67]
Mobile potassium (K_2_O)	By Egner–Riehm–Domingo (A–L) method in a buffer solution (pH 3.7) extraction (lame emission spectrometer)		[67]
Mineral nitrogen (N_min_) (N–NO_3_ + N–NH_4_)	Determined in air-dry samples, 1 M KCl extraction, by flow analysis	Samples (N_min_ and S_min_) are taken every spring after the regeneration of plant vegetation before fertilization with mineral fertilizers. Sampling depth 0–30 and 30–60 cm.	ISO 14256-2:2005
Mineral sulphur (S_min_) (S-SO_4_)	Determined in 1 M KCl extraction by turbidimetric method AOAC-OM-973.57		[68]

**Table 5:** Average precipitation and daily air temperature during the study period.

	**2019**					
	**April**	**May**	**June**	**July**	**August**	**September**
Precipitation, mm	1	29	28	50	100	47
Daily air temperature average, °C	9.0	13.3	21.1	17.1	17.5	12.6
	**2020**					
	**April**	**May**	**June**	**July**	**August**	**September**
Precipitation, mm	6	78	68	67	78	14
Daily air temperature average, °C	6.6	10.3	19.4	17.6	18.4	14.6

## Data Availability

Data is contained within the article.
